# Intestinal microbiota: a new perspective on delaying aging?

**DOI:** 10.3389/fmicb.2023.1268142

**Published:** 2023-11-30

**Authors:** Yuemeng Zhang, Xiaomei Wang, Wujuan Li, Yi Yang, Zhuoxuan Wu, Yuhong Lyu, Changwu Yue

**Affiliations:** ^1^Yan’an Key Laboratory of Microbial Drug Innovation and Transformation, School of Basic Medicine, Yan’an University, Yan’an, Shaanxi, China; ^2^Yan’an University of Physical Education, Yan’an University, Yan’an, Shaanxi, China

**Keywords:** intestinal microbiota, aging, correlation, delay aging, intervention

## Abstract

The global aging situation is severe, and the medical pressures associated with aging issues should not be underestimated. The need and feasibility of studying aging and intervening in aging have been confirmed. Aging is a complex natural physiological progression, which involves the irreversible deterioration of body cells, tissues, and organs with age, leading to enhanced risk of disease and ultimately death. The intestinal microbiota has a significant role in sustaining host dynamic balance, and the study of bidirectional communication networks such as the brain–gut axis provides important directions for human disease research. Moreover, the intestinal microbiota is intimately linked to aging. This review describes the intestinal microbiota changes in human aging and analyzes the causal controversy between gut microbiota changes and aging, which are believed to be mutually causal, mutually reinforcing, and inextricably linked. Finally, from an anti-aging perspective, this study summarizes how to achieve delayed aging by targeting the intestinal microbiota. Accordingly, the study aims to provide guidance for further research on the intestinal microbiota and aging.

## 1 Introduction

The gut has trillions of microorganisms, consisting of archaea, bacteria, fungi, and viruses, in which bacteria are dominant, and they constitute a highly complex and diverse gut microbial kingdom (Sender et al., 2016). Complex connections exist within the intestinal microbial kingdom and between the microbial kingdom and the host. Within the gut microbial kingdom, bacteria use community sensing systems for communication, coordination, and material exchange, colonization competition between bacteria and fungi, the coexistence of bacteria and viruses to promote their diversity and genetic exchange between different microorganisms (Thompson et al., 2016; Ferreiro et al., 2018; Kapitan et al., 2019; Stockdale and Hill, 2021). As an important part of the host, this kingdom acts as a central player in sustaining health, affecting immune cell dynamics, regulating metabolism, and maintaining host dynamic homeostasis and neurological development (Erny et al., 2015; Schluter et al., 2020). Given the important functions of the intestinal microbiota within the host, researchers have focused on the intestinal microbiota and aging, and these studies concerning aging considers the viewpoint of the intestinal microbiota.

The world confronts the crucial challenge of aging. The ageing population is growing much faster than in the past and is expected to reach 1.6 billion by 2050 (Ferrucci et al., 2020). Aging is a sophisticated natural physiological process, and it is not a disease *per se* but an irreversible and time-dependent deterioration of the body’s cellular functions, tissues, and organs with age (Gems, 2014). This deterioration is accompanied by an increased risk of many diseases, such as cardiovascular, pulmonary, and neurodegenerative diseases (Donato et al., 2018; Hou et al., 2019; Cho and Stout-Delgado, 2020). Since the publication of the seven pillars of aging, the latest published hallmarks driving aging have expanded to twelve as aging research has progressed, including dysbiosis, epigenetic alterations, chronic inflammation, stem cell exhaustion, loss of proteostasis, genomic instability, cellular senescence, and telomere wear (Kennedy et al., 2014; López-Otín et al., 2023). Regulation of aging is made possible by accentuating or intervening on driver hallmarks (López-Otín et al., 2023). For example, T-cell dysregulation induces an accumulation of circulating cytokines *in vivo*, resembling chronic inflammation, with significant upregulation of senescence-associated genes (especially *Cdkn1a*) and the emergence of aging-associated phenotypes involving metabolic, muscular, and cardiovascular aspects, which are prevented by treatment with the tumor necrosis factor-α (TNF-α) inhibitor etanercept (Desdín-Micó et al., 2020; López-Domínguez et al., 2021). The anti-inflammatory drug canakinumab, an anti-interleukin-1β (IL-1β) antibody, reduced the incidence of hypertension, diabetes, and lung cancer, providing positive support for further anti-aging research (Ridker et al., 2017). Metformin regulation of aging involves intervention in multiple driver hallmarks, including attenuation of genomic instability, inhibition of inflammation, improvement of nutrient sensing, reduction of cellular senescence, reduction of telomere shortening, and modulation of mitochondrial function (Kulkarni et al., 2020). In clinical trials of metformin, the effectiveness of metformin treatment against targeted aging is supported by a reduction in early mortality associated with several diseases, including cardiovascular disease, cancer, and diabetes (Mohammed et al., 2021). As age-related intestinal microbiota changes are mentioned among the hallmarks of aging, it means that intervening in the intestinal microbiota to achieve anti-aging is possible. Anti-aging related benefits from remodeling the intestinal microbiota have been demonstrated in experimental models of African turquoise killifish, *Caenorhabditis elegans*, and mice (Smith et al., 2017; Kim et al., 2022; Zhang J. et al., 2022). A clinical trial on the effects of fecal microbiota transplantation (FMT) on aging has been initiated (NCT05598112).

We start with the phenomenon of changes in the intestinal microbiota during human aging to gain insight into the link between the gut microbiota and aging. Importantly, we summarize the specific mechanisms and supporting evidence for remodeling the intestinal microbiota and thereby delaying aging from an anti-aging perspective.

## 2 Intestinal microbiota changes during aging

The intestinal microbiota is not static throughout the human lifetime and has a space–time specificity. It co-evolves with the host with age and is affected by both internal aspects such as genetics and external ones such as lifestyle and geography. The progression of the intestinal microbiota has been divided into several stages, namely, neonatal, infant, teenage, adult, aging, and extreme aging (Kundu et al., 2017). The structural components of the intestinal microbiota are characterized at each stage and influence and reinforce each other; these factors include the effects of infancy on the microbiota, including family lifestyle, geographic location, and antibiotic exposure, which also persist into adulthood (Martin et al., 2016).

The components of the intestinal community among healthy adults are generally constant, in which Bacteroidetes and Firmicutes are the dominant bacteria (Wu et al., 2011; Lim et al., 2014). Based on the analysis of the fecal sequencing data of subjects from various countries and by using genus-level clustering and classification methods (e.g., Jensen–Shannon distance and partitioning around medoid), followed by cluster evaluation and statistical analysis, the gut can be divided into the following types based on the main genus: *Bacteroides* (enterotype 1), *Prevotella* (enterotype 2), and *Enterobacteriaceae* (enterotype 3), where enterotype 3 is still controversial, and the Asian enterotype 3 is probably *Ruminococcus* (Arumugam et al., 2011; Wu et al., 2011; Lim et al., 2014; Liang et al., 2017). Geographical differences exist between gender and intestinal bacterial alpha diversity in adults. Based on the analysis of the relationship between gender and intestinal bacteria alpha diversity among a cohort of adults from four geographic regions, the alpha diversity was greater in young women than in men from the United States, United Kingdom, and Colombia, but no association was noted in the Chinese group, and a related study in Japan did not find gender differences in microbiota alpha diversity (de la Cuesta-Zuluaga et al., 2019; Takagi et al., 2019). A comprehensive study of data from 27 analyses of healthy elderly people’s intestinal microbiota revealed higher microbial alpha diversity, variability, and inter-individual variation compared with the youth (Badal et al., 2020).

Several studies have indicated that the intestinal microbiota is significantly altered in the elderly compared to adults, with dysbiosis. As highlighted by the result of recent findings, elderly people’s gut microbiota almost always shows a high abundance of Proteobacteria and Bacteroidetes and a decline in Firmicutes at the phylum level (Odamaki et al., 2016; Salazar et al., 2019; Badal et al., 2020; Herzog et al., 2021; Shimizu et al., 2022). The variations below the phylum levels differ across various studies possibly because of the geographical location, dietary habits, comparison methods, and differences in the age of subjects in different studies. Repeated variations mainly included the relative abundance of *Lachnospiraceae*, *Bacteroidaceae*, *Faecalibacterium*, and *Bifidobacterium* decreased (Odamaki et al., 2016; Salazar et al., 2019; Badal et al., 2020; Herzog et al., 2021; Shimizu et al., 2022). In comparison with adults, beneficial microorganisms decreased and parthenogenic anaerobes increased in the elderly, and the cumulative abundance of most *Lachnospiraceae*, *Bacteroidaceae,* and *Ruminococcaceae* in the overall core microbiota decreased with age (Biagi et al., 2016). Studies on gut microbiota are mainly based on 16S rRNA and macroeconomic analysis of feces. Leite et al. collected duodenal aspirates from 251 subjects of different ages and found reduced microbial diversity, declined relative abundance of Bacteroidetes, and elevated abundance of Proteobacteria, *Enterobacteriaceae*, and *Lactobacillaceae* in the elderly (Leite et al., 2021).

The intestinal microbiota is closely correlated with aging. Even a method for predicting host age based on a taxonomic profile of microbial communities has been developed using cross-sectional research datasets and deep learning, in which *Bacteroides*, *Eubacterium*, and *Bifidobacterium* are the most useful bacteria for prediction, and this microbiota aging clock tested on external data showed that the mean absolute error of this architecture reached 5.91 years (Galkin et al., 2020).

## 3 Relevance of the intestinal microbiota to aging

Alterations in the intestinal microbiota with age have been demonstrated. The correlation between the intestinal microbiota and organismal aging is of research interest. Currently, the causative relationship between altered intestinal microbiota and aging is still debated, i.e., it has not been determined whether aging is a result of the evolution of its intestinal microbiota or a cause of microbiota changes.

### 3.1 Altered intestinal microbiota affects aging

The vital contribution of age-related intestinal microbiota changes is mentioned in the latest release of driving hallmarks of aging (López-Otín et al., 2023). When the microflora of aged mice was transferred to young mice, the intestinal barrier integrity of young mice was broken, bacterial translocation increased, and bacterial products leaked, thus affecting systemic and tissue inflammatory responses, age-related neurodegeneration; moreover, retinal function degeneration occurred, and some aging features appeared (Parker et al., 2022). Intestinal microflora from young and aged mice was engrafted into young germ-free (GF) mice by FMT. In comparison with young recipients, aged mice have significantly lower alpha diversity in fecal samples, are more deprived of short-chain fatty acid (SCFA)-producing taxa, behave in a more depression-like manner, produce cognitive decline, and develop an aging-related phenotype (Lee et al., 2020). Sirtuin6 (SIRT6) is associated with mammalian lifespan, regulates chromatin structure and DNA repair, maintains genomic stability, and knocks down the SIRT6 gene to construct a mouse model of premature aging (Mostoslavsky et al., 2006). Can the intestinal microbiota of prematurely aged mice drive aging? Experiments with antibiotic cocktail therapy on the intestinal microbiota of prematurely aged mice resulted in the reduced expression of aging markers (e.g., p16, p21) and inflammatory responses in mice (Xu et al., 2023). The fecal microbiota of prematurely aged mice was transferred to wild-type (WT) mice, and the transferred mice showed dysbiosis, reduced alpha diversity, disrupted intestinal barrier, hair coarseness, fat loss, and visceral senescence, and found that *Enterobacteriaceae* translocation may play a significant role in gut microbiota-mediated senescence (Xu et al., 2023). These studies support that the aging gut microbiota alone can cause age-related deleterious effects in the organism.

Altered intestinal microbiota can influence other aging-driving processes and collectively contribute to aging. For example, changes in the intestinal microbiota are closely linked to chronic inflammation, and even in the absence of any damage, young individuals receiving aging microbiota transplants enhance systemic inflammation and increase the host levels of systemic pro-inflammatory cytokines (Spychala et al., 2018). Researchers transplanting intestinal microbiota from aged and young mice separately to GF mice showed that mice receiving aged microbiota experienced leakage of bacterial components and increased expression of inflammatory markers *in vivo*, confirming that microbiota from aged mice can induce host inflammatory responses (Fransen et al., 2017). The altered gut microbiota can affect the body’s immune cell status, as in *LysM-cre/Myd88^fl/fl^* mice based on the straightforward connection between the intestinal microbiota and circulating neutrophil aging, which upregulates pro-inflammatory effects (Zhang et al., 2015). Leaky bacterial components drive neutrophil aging through the intestinal Toll-like receptor (TLR)-myeloid differentiation factor 88 (Myd88) pathway, while microbiota depletion dramatically reduces the amount of cycling aging neutrophils and ameliorates inflammation-associated organ damage (Zhang et al., 2015). The intestinal microbiota is extensively connected to macrophages (Mortha et al., 2014). Age-related changes in the intestinal microbiota leads to distal microglia dysfunction, mainly due to leakage of the microbial metabolite N^6^-carboxymethyllysine, which impairs cellular mitochondrial function (Mossad et al., 2022). Host T cells are also affected, with splenic CD4^+^ T cell differentiation is enhanced, possibly due to the leakage of gut microbial derivatives (Fransen et al., 2017). Alterations in the gut microbiota affect inflammation-related pathways, such as the nuclear factor-kappa B (NF-κB) signaling pathway, causing a widespread systemic inflammatory response (Jeong et al., 2015; Kim et al., 2016).

Besides inflammation, DNA damage is one of the hallmarks of aging, and highly conserved mechanisms for detecting and repairing DNA damage are important (White et al., 2015; Yousefzadeh M. et al., 2021). Reduced DNA repair efficiency leads to the accumulation of double-strand breaks (DSBs), which may cause aging (Guedj et al., 2016). Dysbiosis in mice leads to the leakage of lipopolysaccharide (LPS) into the hepatic portal vein, increased generation of inflammatory cytokines IL-1β, TNF-α, and chemokine RANTES in the liver, upregulation of genes related to inflammation, downregulation of genes associated with glucose, cholesterol, and fatty acid homeostasis, and consequently reduced repair of DSBs (Guedj et al., 2020). Stem cell exhaustion is also one of the hallmarks of aging, and the association between intestinal microbiota and stem cell exhaustion is the pivot of the gut microbiota that indirectly drives age (Wanet et al., 2015). Altering the intestinal microbiota of mice through a high-fat diet (HFD) leads to a decrease in hematopoietic stem cells, which can be achieved by transferring feces from mice on HFD to normal mice (Luo et al., 2015). Due to the increased intestinal permeability, leaky microbes and their metabolites directly promote abnormal proliferation and overuse of stem cells by activating Wnt, Notch, and other signal pathways, and impair stem cell–microenvironment interactions, leading to stem cell pool depletion and accelerated host aging (Tan et al., 2019). Leaky gut microbiota metabolites SCFA may bind to G protein-coupled receptors, affecting host stem cell metabolism and interfering with insulin signaling, thereby affecting the integrity and activity of the mitochondrial electron transport chain, resulting in an unbalance between glycolysis and OXPHOS, ROS accumulation, and mitochondrial dysfunction ultimately leading to stem cell misdifferentiation and proliferation, causing stem cell exhaustion (Cheng et al., 2010; Jocken et al., 2017; Ermolaeva et al., 2018). Aging is associated with increased insulin resistance (IR) (Costantino et al., 2016). Altered intestinal microbiota was closely connected to IR, with higher fat body weight and insulin levels in mice receiving feces from aged mice compared to mice receiving feces from adult mice (Binyamin et al., 2020). The analysis of aged mice and macaques showed that altered intestinal microbiota resulted in increased intestinal permeability and bacterial products (such as palmitic acid and LPS) and activation of CCR2^+^ inflammatory monocytes, prompting a strong expression of TNF-α and 4–1BBL by transformed 4BL cells and thus inducing IR (Bodogai et al., 2018). LPS also triggers NLRP3 inflammasome activation, leading to impaired IR and glucose tolerance (Bauernfeind et al., 2016).

The intestinal microbiota undergoes age-related alterations, and its metabolites may change as well. Intestinal microbiota metabolites can be broadly classified according to their source into three categories, including those produced by the conversion of exogenous dietary substrates, those produced by modification of endogenous host compounds, and those synthesized *de novo* by the gut microbiota (Postler and Ghosh, 2017). Among the metabolites produced using exogenous dietary substrates, trimethylamine-converted trimethylamine-N-oxide (TMAO) cycle levels increase as humans and mice age, and the experimental administration of TMAO intervention increases hippocampal CA3 region’s neuronal aging and enhances oxidative stress-driven mitochondrial damage in mice (Li et al., 2018). *In vitro*, TMAO treatment of human umbilical vein endothelial cells (HUVECs) reduces its proliferation, decreases the migration, and enhances the expression of aging markers (Ke et al., 2018). The inhibition of sirtuin1 (SIRT1) production and upregulation of oxidative stress by TMAO both *in vitro* and *in vivo*, followed by p53-p21-retinoblastoma protein (Rb) pathway activation, leads to the increase in acetylation of p21 and p53 (Ke et al., 2018). In TMAO-treated HUVECs and ApoE^−/−^ mice model of aortas, promoting the NLRP3 inflammatory vesicles activates inflammatory responses, inhibits sirtuin3 (SIRT3) and superoxide dismutase-2 (SOD2) expression, induces mtROS accumulation, and upregulates succinate dehydrogenase complex subunit B (SDHB) expression, thereby impairing mitochondrial structure and function (Chen et al., 2017; Wu P. et al., 2020). Increased TMAO in mice brains elevate the NF-κB and inflammatory cytokine levels possibly through neuroinflammation under direct exposure, which is connected with impaired cognition (Brunt et al., 2021). Therefore, aside from triggering oxidative stress and mitochondrial damage to induce cellular senescence, TMAO is also associated with inflammatory responses. Increased TMAO indirectly promotes aging progression and accelerates the occurrence of aging-related diseases, such as neurodegenerative and cardiovascular conditions (Zhang L. et al., 2022). Gut microbial metabolites are also involved in driving aging and accelerating the occurrence of certain age-related pathologies.

**Figure 1:**
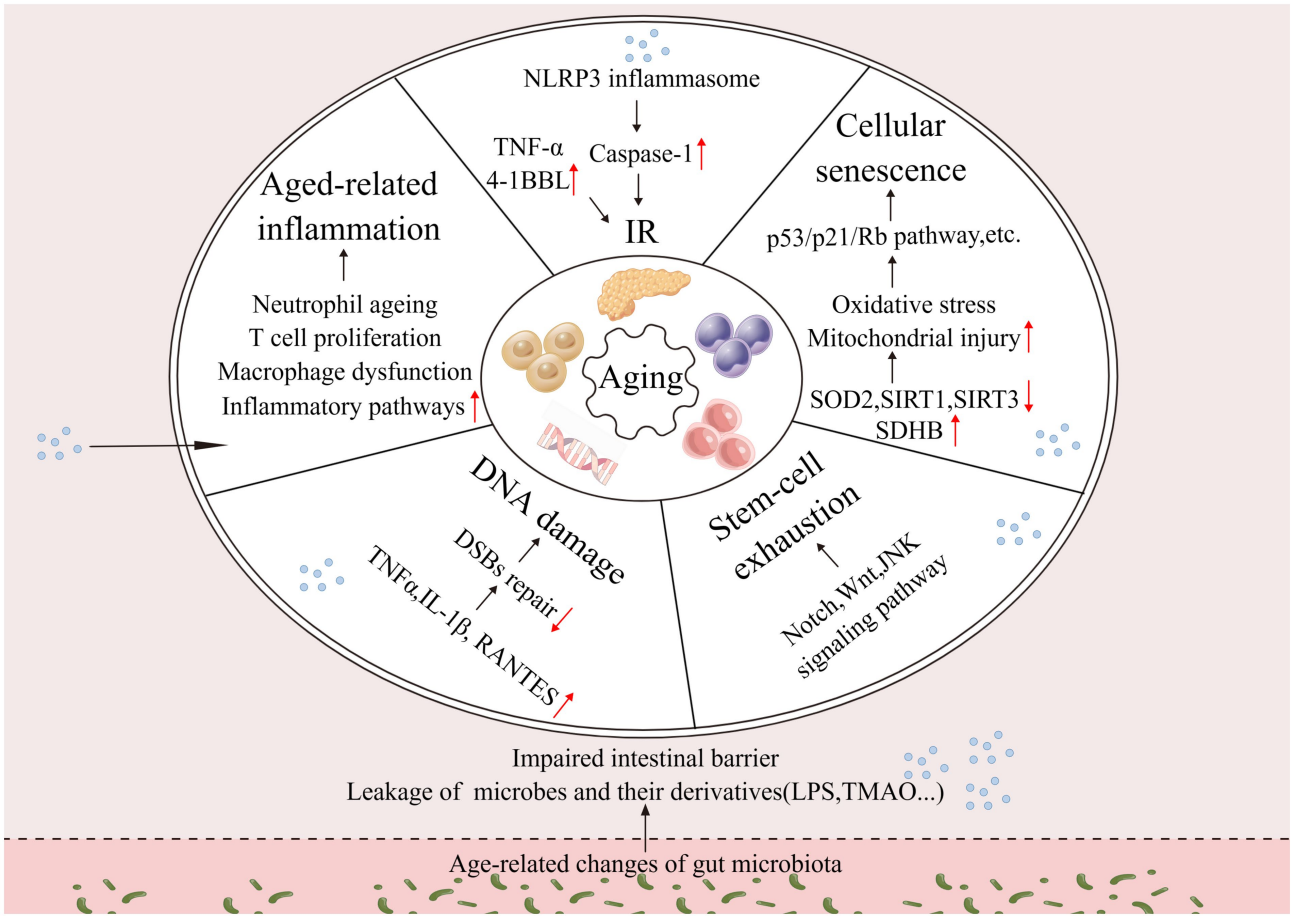
Changes in the intestinal microbiota drive aging. Changes in the gut microbiota affect host inflammation, insulin resistance, DNA damage, stem cell exhaustion, cellular senescence, and other processes that together drive aging (↑: increase; ↓: decrease).

Aging does not begin at a specific age; it is a gradual process, with changes gradually over time. During aging, the gut microbiota undergoes alterations, and its mediated increase in intestinal permeability leads to chronic inflammation, IR, decreased efficiency of DNA repair, stem cell depletion, and cellular senescence of the organism. Altogether, these processes contribute to aging (Figure 1). The drivers of aging are interconnected and interact with each other and deserve to be explored in depth.

### 3.2 Altered intestinal microbiota caused by aging

**Figure 2:**
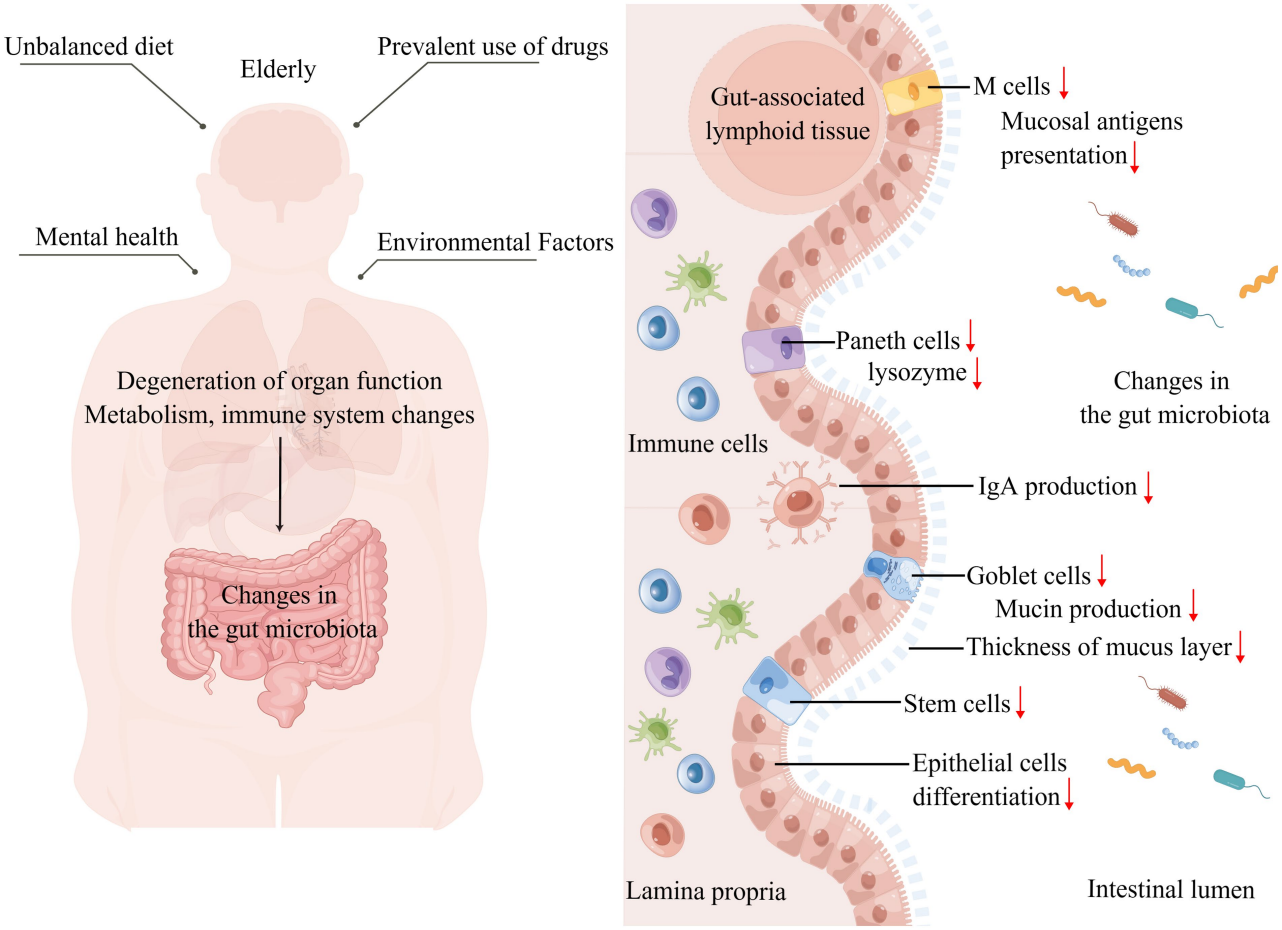
Altered intestinal microbiota caused by aging. Left: The unique diet, mental health, drug use, and living environment of the elderly affect their gut microbiota shaping, while organ degeneration, physical function decline, metabolic changes, and immune system specific changes that accompany aging affect the gut microbiota. Right: Focusing on the effects of aging on the gut, the intestinal barrier is damaged, the mucus layer becomes thinner, increasing the direct contact between the intestinal microbiota and microvilli, the number of intestinal epithelial cells, M cells, and Paneth cells decreases, the number and function of immune cells in the lamina propria of the intestine change, IgA production decreases, and the intestinal immune homeostasis is broken, thus affecting the gut microbiota (↑: increase; ↓: decrease).

Currently, aging is inevitable among humans; aging is a complex physiological process, and certain species do not show signs of aging as they age (Holtze et al., 2021). Considering that aging is inevitable in humans, alterations in the intestinal microbiota due to organismal aging become possible. The living environment, drug use, mental changes, and metabolism of the elderly may affect the intestinal microbiota (Figure 2). A study investigating the intestinal microbiota of 166 elders residing in five nursing homes (NH) respectively found that it was influenced by the duration of residence, with an enhanced abundance of pathogenic microbes during the first 6 months of residence, decreased anti-inflammatory microbes after 6 months, and dysbiotic microbiome pattern occurring after 1 year of residence, characterized by decreasing symbiotic species and increasing pathogens, after which it shows relative stability over time (Haran et al., 2021). The analysis of specific influencing factors revealed that NH site, diet, and drug use affect the gut microbiota, in which psychoactive, anti-hypertensive, metabolic, and dementia drugs have the greatest effect on microbiome composition (Haran et al., 2021). A study on the fecal microbiota of 76 elderly inpatients showed that their microbiota beta diversity was markedly different from outpatients, the number of medications used was negatively related to microbiota biodiversity, and single taxon abundance was strongly correlated with antipsychotics, proton pump inhibitors, and antidepressants (Ticinesi et al., 2017).

Diet is essential for molding the gut microbiota (Zmora et al., 2019). Based on a survey, the dietary nutritional needs of the elderly seem to be unmet, and malnutrition, especially subclinical vitamin and trace element deficiencies and protein-energy malnutrition, are prevalent among the elderly (Borgström Bolmsjö et al., 2015; Hoogendijk et al., 2018). The oral health status of the elderly and the weakened gastrointestinal tract and loss of appetite can affect dietary intake and thus reshape their intestinal microbiota. The oral condition of the elderly is worrisome, and many elderly people experience difficulties in chewing and swallowing, decreased salivary gland function, tooth loss, dry mouth syndrome, and poor oral hygiene (Gibney et al., 2021). Gum disease and tooth loss are correlated with less alpha diversity of colonic bacteria, and the more tooth loss, the lower the relative abundance of *Faecalibacterium* (Xu et al., 2021). Aging decreases gastrointestinal tract dynamics, decreases intestinal tone, and slows intestinal motility. Rhythmic intestinal motility cannot be achieved without the interstitial cells of Cajal (ICCs), which act as pace-making cells in the gastrointestinal system. Connexin43 (Cx43) is an important protein that is responsible for intercellular signaling and is widely present between ICCs and/or smooth muscle cells (Foong et al., 2020). In aged mice with gastrointestinal losses of enteric neurons and ICCs, Cx43 protein expression is reduced; similarly, in the colonic muscle layer of the elderly, the level of ICCs-related c-kit protein is lower, and the key components involved in neurotransmitter syntheses such as choline acetyltransferase and neuronal nitric oxide synthase expression are disturbed (Sun et al., 2018). Problems with gastrointestinal function are directly linked to nutrient absorption, and mice fed a nutrient-deficient diet have significantly more diverse intestinal microbiota with altered composition (Håkansson et al., 2021).

The bidirectional communication of organs and intestines have been studied widely, and the brain–gut, liver–gut, and heart–gut axes have been successively demonstrated (Mohajeri, 2019; Zuo et al., 2019; Ohtani and Hara, 2021). Does the degeneration of organ function in the elderly affect the shaping of their intestinal microbiota? Taking the brain-gut axis as an example, the elderly have increased brain atrophy, altered functional whole brain dynamics, altered modular connectivity, altered brain nerve cell structure, impaired microstructure, increased neurodegeneration, decreased brain glucose metabolism, and brain iron accumulation, etc.; the accumulation of degenerative brain processes affects their brain function with cognitive decline, decreased responsiveness, and behavioral impairment (Pannese, 2011; Grubić Kezele and Ćurko-Cofek, 2020; Indahlastari et al., 2020; Li X. et al., 2020; Chen X. et al., 2021; Escrichs et al., 2021; Reas et al., 2021). Changes in cognitive abilities lead to corresponding changes in microbiota characteristics. The Mindful Awareness Program intervention in patients with mild cognitive impairment (MCI) improved their cognitive ability, and the changes in cognitive ability led to alterations in intestinal *Ruminocococeae*, *Coprococcus*, *Ruminococcus*, *Enterobacteriaceae*, *Fusobacterium*, *Phascolarctobacterium*, and *Parabacteroides* abundance, in which *Ruminococcus* is related to Memory Domain, Semantic Fluency Span, Recognition Trial, and Digit Span Backward (Khine et al., 2020). The degeneration of brain function in the elderly is correlated with the risk of several diseases such as stroke (Grubić Kezele and Ćurko-Cofek, 2020), delirium (Bugiani, 2021), Parkinson’s disease, Alzheimer’s disease, and chronic subdural hematoma (Bin Zahid et al., 2018). These diseases are inextricably linked to the gut microbiota, such as gastrointestinal complications after stroke, ischemic brain tissue and microglia releasing inflammatory molecules, cellular debris, and activated vagal, which can induce gut motility disorders, barrier damage, and dysbiosis (Ritzel et al., 2018; Ye et al., 2021). The bacterial overgrowth of Bacteroidetes and decreased species diversity are signs of dysbiosis after stroke (Singh et al., 2016).

In the gut, which is in intimate touch with the intestinal microbiota, altered gut barriers in aging contribute to shaping the gut microbiota. The mucus layer consists mainly of highly glycosylated mucins produced by cupped cells, which provide nutrients and adhesion sites for the intestinal microbiota (Sicard et al., 2017). The thickness of the colonic mucus layer was found to decrease by approximately six-fold in aged mice compared with younger mice, implying a greater penetration of bacteria in the lumen and increased contact with villus epithelium, and these changes are likely to severely affect the microbiota (Sovran et al., 2019). Aged mice have increased intestinal epithelial cell apoptosis, decreased regenerative capacity, and reduced expression of Wnts in intestinal stem cells (ISCs), Paneth cells, and subepithelial mesenchymal cells, which compromise intestinal integrity (Moorefield et al., 2017; Nalapareddy et al., 2017; Cui et al., 2019). At the molecular level, the secretion of regenerating islet-derived protein, Ang4, CXCL8, lysozyme, IgA and other factors is reduced in the intestinal lumen, leading to alterations in the intestinal microbiota (Sovran et al., 2019; Xiao et al., 2019, 33).

The immune system undergoes alterations in the elderly, including reduced responsiveness to new antigens, degeneration of primary lymphoid organs, reduction of naive cells, decreased lymphopoiesis, accumulation of dysfunctional memory cells, and a state of chronic inflammation (Ciabattini et al., 2018; Aiello et al., 2019). In gut mucosal immunity, aging-related immune alterations are intimately linked to the reshaping of the intestinal microbiota. Microfold cells (M cells) present in follicle-associated epithelia (FAE) are responsible for the uptake and transcytosis of mucosal antigens in the intestinal lumen to gut-associated lymphoid tissue (Kobayashi et al., 2019). In aged mice, M cell density was significantly reduced, the expression of Spi-B, an intrinsic Ets transcription factor, and C-C motif chemokine ligand 20 were impaired, which impeded the maturation and differentiation of M cells, and the senescence-related changes in Paneth cells indirectly affected M cell differentiation and the shaping of the intestinal microbiota (Kobayashi et al., 2013; Donaldson et al., 2021). The combined production of secreted IgA by plasma cells and epithelial cells promotes intestinal homeostasis through various mechanisms, including blocking the bacterial invasion into the gut, promoting symbiosis between bacteria, and controlling inflammatory responses to beneficial microorganisms, etc., (Nakajima et al., 2018; Pabst and Slack, 2020). The transcription of intestinal IgA-related genes was significantly downregulated in aged mice (Sovran et al., 2019). However, in another study, no significant difference was observed in the IgA concentrations between the elderly and adult groups, but a reduced IgA response was observed in the gut environment of the elderly, which may have increased the abundance of potentially pathogenic bacterial taxa (Sugahara et al., 2017). CD4^+^ T cells, which make up the bulk of T cells in the intestinal mucosa (LP), are essential for sustaining gut homeostasis and antimicrobial defense. The comparison of LP CD4^+^ T cell function and phenotype from young and elderly individuals using isolated human small intestinal cell models revealed increased levels of spontaneous cell death in CD4^+^ T cells, decreased expression of co-suppressor molecules, reduced frequency of Th17 in Th cell subpopulations, and defective responses of Th1 and Th17 cells to gut-associated bacterial species in aged samples, changes that may affect the intestinal microbiota (Dillon et al., 2020).

Overall, many factors are both the cause and the result of aging. Immune aging and chronic inflammatory states promote aging and are the consequence of body aging, and the intestinal microbiota is no exception. Regarding the causal debate between aging and intestinal microbiota, we suggest that they are mutually causal, mutually influencing, mutually promoting, and inseparable.

## 4 Delaying aging through the gut microbiota

Aging measurements include physiological and functional measurements (walking speed, pulse wave velocity, maximal oxygen uptake, and organ function), molecular biomarker measurements (DNA methylation data) (Fransquet et al., 2019). Aging is considered a principal risk element for disease, and interventions for aging focus on both its progressive and deleterious aspects (Ros and Carrascosa, 2020). Safe ways are being developed to delay aging progression in order to minimize the harmful consequences of aging. Delaying aging mainly works on the driving process of aging to achieve essential interventions that slow down organismal physiological and functional degeneration and the onset of age-related disorders (Justice et al., 2018; López-Otín et al., 2023). Measures to delay aging include maintenance of macromolecular homeostasis, regulation of epigenetic and mitochondrial function, inhibition of immune aging, removal of senescent cells, and cellular reprogramming, etc., (Simpson et al., 2021; Yousefzadeh M. J. et al., 2021; Moskalev et al., 2022). In recent years, studies on reshaping the intestinal microbiota to delay aging have been increasing, mainly through FMT, probiotics, various dietary patterns, and natural compounds (Table 1).

**Table 1:** Summary of various interventions that modulate the gut microbiota to delay aging in different models.

	Intervention	Species/model	The gut microbiota-related changes	Outcomes	Ref
FMT	Transplanted the fecal microbiota from young (3 months) to aged (18 months and 24 months) mice	Mice	↑Bifidobacteria, Eubacteria, and *Akkermansia* Improves colony lipid and vitamin (B7 and B9) metabolism	•Reverses age-related deleterious changes in the gut, brain and retina	Parker et al. (2022)
	Transplanted the fecal microbiota from young (5 weeks old) to aged (12 months and 25 months) mice	Mice	↑*Muribaculaceae, Bacteroidaceae, Lactobacillaceae,* and *Prevotellaceae*	•Increases muscle thickness and body recovery	Kim et al. (2022)	Transplanted the fecal microbiota from young (5 weeks old) into aged (42 weeks old) mice	Mice	↑*Bifidobacterium* and *Ruminococcaceae* Improving flora metabolism	•Restores ovarian function	Xu L. et al. (2022)	Transplanted the fecal microbiota from young (6 weeks old) to middle-aged (9.5 weeks old) fish	African turquoise killifish	↑Species diversity, *Carnobacterium, Arthrobacter, Exiguobacterium, Planococcus, Psychrobacter, Enterococcus, and Halomonas*	•Delays behavioral decline	Smith et al. (2017)	Transplanted the fecal microbiota from centenarians into mice (11 months)	Mice	↑Alpha Diversity, *Lactobacillus, Bifidobacterium， Roseburia, Faecalibacterium, Ruminococcus,* and *Coprococcus*	•Senescence-associated reduces in lipofuscin and β-galactosidase •Intestinal villi development	Chen et al. (2020)	Oral FMT capsules	Human	/	/	/
Probiotics	*Lactobacillus plantarum* GKM3 intervention SAMP8 (24 weeks old) until death	Mice	/	•Alleviates age-related cognitive impairment •Maintains memory and learning	Lin et al. (2021)
	*Lactobacillus paracasei* PS23 and Heat-killed PS23 interventions in D-gal-induced aging model for 9 weeks separately	Mice	↑Firmicutes, Bacteroidetes, and Tenerictes ↓*Bifidobacterium*, *uncultured_Bacteroidales_bacterium*, *and Coriobacteriaceae _UCG_002*	•Delays age-related cognitive decline in mice •Improves motor function •Regulates age-related genes in aging mice	Cheng et al. (2022)	*Lactobacillus paracasei* PS23 intervention SAMP8 (16 weeks old) 12 weeks	Mice	↑Lactobacillales and Pseudomonadales ↓*Lachnospiraceae*_UCG_001 and IgA	•Reduces aging scores •Delays age-related cognitive decline	Huang et al. (2018) and Chen L. H. et al. (2021)	*Lacticaseibacillus rhamnosus* probio-M9 intervenes throughout the life cycle	*C. elegans*	/	•Delays the loss of exercise capacity •Decrease in lipofuscin accumulation	Zhang J. et al. (2022)	Heat-killed *Lactobacillus paracasei* D3-5 intervention mice (>79 weeks old) 10 weeks	Mice	↑Verrucomicrobia and Mucin ↓Firmicutes	•Restores physical and cognitive function •Prevents metabolic disorders	Wang et al. (2020)	*Bifidobacterium longum* intervention D-gal-induced aging model for 9 weeks	Mice	Regulates the overall metabolism of the gut microbiota, especially the arginine and linoleic acid metabolic pathways	•Reverses learning and memory impairments associated with aging •Prevents a decline in activity levels	Xiao et al. (2021)
	*Lactobacillus plantarum* 69-2 with the GOS combined intervention in D-gal-induced aging model for 6 weeks	Mice	↑F/B ratio, *Akkermansia*, *Lactobacillus*, *Blautia*, *Mucispirillum*, *Butyricicoccus*, SCFA ↓*Bacteroides*, *Helicobacter*, and *Escherichia-Shigella*	•Reduces levels of aging-related genes •Increases liver and spleen coefficients	Wang et al. (2021)
Diet	Methionine restriction interventions in D-gal-induced aging model for 8 weeks	Mice	↑*Lachnospiraceae*, *Turicibacter*, *Roseburia*, *Intestinimonas*, *Tyzzerella*, *Rikenellaceae*, *Ruminococcaceae*, SCFA	•Prevents age-related decline in learning and memory function •Alleviates anxiety-like behaviors	Xu Y. et al. (2022)
	Wushen intervention SAMP8 (12–16 weeks old) 15 weeks	Mice	↑*Ruminococcus* and *Butyrivibrio* ↓*Lachnoclostridium*, *Ruminiclostridium*, *Eubacterium coprostanoligenes*, and *Intestinimonas* improving flora metabolism	•Alleviates aging-related loss of muscle mass and fat content •Improves learning and memory capacity •Reduces cognitive decline	Zhang et al. (2021)
Natural compounds	Taxifolin intervention in D-gal-induced aging model for 6 weeks	Mice	↑F/B ratio, *Enterorhabdus*, *Clostridium*, *Bifidobacterium*, and *Parvibacter*	•Reverses neuronal damage in brain tissue associated with aging •Reduces spatial learning and memory impairment	Liu et al. (2021)
	Phlorizin intervention in D-gal-induced aging model for 5 weeks	Mice	↑*Lactobacillus* and *Bacteroides* ↓F/B ratio	•Delays cognitive impairment •Mitigates organ coefficient decline in aging mice	Chen et al. (2022)
	Lemon polyphenols intervention SAMP1 (9 weeks old) until death	Mice	↑F/B ratio ↓*Lactobacillus*	•Delays increase in aging-related scores and motor atrophy	Shimizu et al. (2019)
	Icariin intervention in young (8 weeks old) and old (24 months old) mice for 15 days respectively	Mice	↑*Butyrivibrio* and *Mucispirillum* ↓Alpha Diversity, *Akkermansia* and *Alistipes*	•Improves motor learning and coordination in aged mice •Upregulates some aging-related genes	Li et al. (2021)	RNEA intervention in D-gal-induced aging model for 12 weeks	Mice	↑*Staphylococcaceae* and *Corynebacteriaceae* ↓*Ruminococcaceae* and *Oscillospiraceae*	•Improves aging-related memory loss	Guo et al. (2022)

↑: increase; ↓: decrease.

### 4.1 FMT

FMT is the most direct way to interfere with host gut microbes; FMT can transfer the whole microbiota and make up for the limited nature of probiotics, which are mainly used clinically in the treatment of gastrointestinal diseases, and serious adverse effects are uncommon (Quraishi et al., 2017; Green et al., 2020). Whether aging can be delayed by FMT needs to be looked into. Preclinical studies have focused on mice models. Aged mice receiving the intestinal microbiota transplantation from young mice were enriched with *Bacteroidaceae*, *Muribaculaceae*, *Prevotellaceae,* and *Lactobacillaceae* in their microbiota, and the transplanted aged mice had increased muscle thickness and recovered physical fitness for an improved aging phenotype (Kim et al., 2022). The transfer of aged gut microbiota to young mice by FMT accelerated its age-related deleterious effects, while aged mice receiving young mice were enriched for Bifidobacteria, Eubacteria, and *Akkermansia*, promoted mucin generation and epithelial barrier integrity, declined LPS levels, and reversed deleterious effects in the brain, retina, and gut (Parker et al., 2022). Similarly, older mice receiving gut microbiota from younger mice tended to be rejuvenated in microbiota composition, with increased levels of commensal bacteria (e.g., *Bifidobacterium* and *Ruminococcaceae*), improved flora metabolism, inhibited aging-associated mTOR signaling pathways, restored ovarian function, and improved fertility in aged mice (Xu L. et al., 2022). In African turquoise killifish, middle-aged subjects were transplanted with a younger gut microbiota after the antibiotic intervention, resulting in significant changes in their community structure; this process induced long-term beneficial systemic effects after transplantation, delayed behavioral decline, and increased the activity similar to the performance of young fish (Smith et al., 2017). In addition to receiving the intestinal microbiota of young individuals, the gut microbiota of centenarians was transferred into mice (Chen et al., 2020). The intestinal microbiota of a long-lived population is distinct from that of the elderly and has special characteristics (Wang et al., 2022). Mice receiving the gut microbiota of centenarians had greater alpha diversity, richer probiotic genera and SCFA-producing genera, and less *Bilophila wadsworth* compared with those receiving the gut microbiota of older adults (Chen et al., 2020). Current animal experiments essentially involve receiving donors of the gut microbiota by oral gavage after antibiotic treatment, achieving successful transfer by increasing the frequency and duration of FMT interventions, and detecting the corresponding aging-related markers by performing them at the end of the cycle. However, the selection of an appropriate FMT dosing regimen for both disease treatment and anti-aging studies needs to consider long-term intestinal microbiota monitoring and the persistence of the positive effects on the organism. A clinical trial on the effects of FMT on aging has been initiated and is currently in Early Phase 1 (NCT05598112), from which satisfactory results are expected.

### 4.2 Probiotics

The definition of probiotics is “living microorganisms that, when used in moderation, provide health benefits to the host” (Hill et al., 2014). Moderate intake can balance and optimize the intestinal microbiota, thus providing favorable effects in the body, of which *Lactobacillus* is the most common (Zhang et al., 2020). *Lactobacillus plantarum* GKM3 (GKM3) extends the lifespan of senescence-accelerated mouse prone 8 (SAMP8) mice, especially in females (Lin et al., 2021). GKM3 intervention prevents amyloid accumulation in their brains, delays neuronal damage caused by aging in the hippocampus, alleviates age-related cognitive impairment, contributes to age-related memory maintenance and learning enhancement, and delays the aging process (Lin et al., 2021). D-galactose (D-gal) is widely used in experimental studies for evoking pathological changes similar to natural aging in animals. *Lactobacillus paracasei* PS23 (PS23) treatment of D-gal-induced mice leads to alterations in the intestinal microflora composition, enriching the families Bacteroidetes, Firmicutes, and Turners and decreasing the relative abundance of *uncultured Bacteroidales bacterium*, *Clostridia*_UCG_002, and *Bifidobacterium* (Cheng et al., 2022). These microbial-related changes may play a beneficial role, where PS23 may increase 5-hydroxytryptamine (5-HT) levels in the hippocampus, improve spatial memory impairment and motor function, and modulate age-related genes in aging mice (Cheng et al., 2022). Similarly, when PS23 intervention in SAMP8 mice is initiated at the early phase of aging, it delays aging performance, reduces aging scores, and delays the advancement of cognitive impairments associated with aging (Huang et al., 2018). Based on the exploration of the intestinal microbiota of SAMP8 mice, PS23 treatment enhanced Lactobacillales and Pseudomonadales, reduced *Lachnospiraceae*_UCG_001, and alleviated the age-related decrease of IgA in the intestine (Chen L. H. et al., 2021). *Lacticaseibacillus rhamnosus* Probio-M9 (Probio-M9) delayed the decline in motility and significantly reduced lipofuscin accumulation in adult *C. elegans*, and significant increases in amino acids, sphingolipids, galactose, and fatty acids in its microbial metabolites may play a role (Zhang J. et al., 2022). Impaired gut integrity is critical for the participation of microbiota in aging, heat-killed *Lactobacillus paracasei* D3-5 (D3-5) reduces age-related intestinal leakage and inflammation, prevents metabolic dysfunction, and enhances physical function in aged mice (Wang et al., 2020). In terms of mechanism, lipoteichoic acid plays an important role, and D3-5 improves the intestinal microbiota of aged mice, showing a remarkable increase in the metabolically beneficial Verrucomicrobia (Wang et al., 2020).

In addition, by using *Bifidobacterium longum*, a representative of host-microbe coevolution, for the treatment of D-gal-induced mice, the researchers found that strains with specific genotypes (genomic locus 278, RG4-1, with an AGT allele on FJSWXJ10M2) modulated the overall metabolism of the mice gut microbiota, mainly affecting the arginine and linoleic acid metabolic pathways, enhancing bacterial arginine enrichment, reversing the learning and memory impairment associated with senescence, and preventing a decrease in activity levels in senescent mice (Xiao et al., 2021). The definition of a prebiotics is “a substrate that is selectively utilized by host microorganisms, conferring a health benefit” (Gibson et al., 2017). Prebiotics mostly work better in combination with probiotics, even more effective than probiotics alone. For example, *Lactobacillus plantarum* 69-2 coupled with galactooligosaccharides (GOS) could regulate the intestinal flora of D-gal-induced mice, prevent the decrease in Shannon index, affect the flora structure, increase the level of SCFA and the thickness of intestinal mucosa, protect the intestinal barrier, and then activate the adenosine 5′-monophosphate-activated protein kinase/Sirtuin-1 (AMPK/SIRT1) pathway, reduce the level of aging-related genes (Wang et al., 2021).

In terms of the use of probiotic therapy to delay aging, no clinical trials with large sample size have been conducted, and sufficient evidence-based proof remains lacking. As a functional food, a reasonable dosing regimen is needed to achieve the desired effect, the dependence on the benefits generated needs to be determined, and the health risks associated if the treatment is discontinued or taken for a long time need to be determined. In addition, the issue of colonization resistance needs to be further studied, including the risk of translocation of probiotics and subsequent serious infections such as bacteremia and sepsis, especially in immunocompromised and pediatric populations (Dani et al., 2016; Bafeta et al., 2018). Enhanced colonization can be achieved through enhanced niche competition, genetic modification, and surface modification to construct engineered probiotics or by considering the use of postbiotics (Piqué et al., 2019; Huang et al., 2022). The assessment of colonization cannot be limited to stool testing alone, but intestinal mucosal testing also needs to be considered. Probiotic colonization differs across different individuals, which is related to the host’s microbiome characteristics; however, the gut microbiota varies greatly among individuals, especially in the elderly, so individualized interventions are recommended to select appropriate therapies by assessing the characteristics of the organism (Zmora et al., 2018).

### 4.3 Diet

Diet is important in determining the intestinal microbiota, and delaying aging is more reliable through some dietary patterns, especially calorie restriction (CR). CR refers to a 20–50% induction of energy intake by the body without malnutrition, and CR delays the advancement of aging and aging-related conditions based on many experimental models; even clinical trials have similarly supported the role of CR on aging (Belsky et al., 2017; Kwon and Belsky, 2021; Ramaker et al., 2022). However, CR delays aging mainly by mechanisms that regulate energy metabolism, decline oxidative damage and inflammation, modulate nutrient-sensing pathways, and enhance cytoprotection, as well as by epigenetic factors (Hearn et al., 2019; Hwangbo et al., 2020). Although the effects of CR on the intestinal microbiota have been reported, a direct proof that it exerts an aging-delaying effect by interfering with the microbiota has not been provided (Tan et al., 2019; Kurup et al., 2021). The CR remodeling of the intestinal microbiota mediates improvements in organismal metabolism that may contribute to delaying aging. GF mice transplanted with the intestinal microbiota from CR mice showed enhanced glucose tolerance and insulin sensitivity, increased fat browning, and reduced fat volume and mass mainly due to the increased arabinose dependence of some bacterial groups during nutrient deprivation and reduced LPS caused by the low production of bacterial enzymes essential for lipid A biosynthesis (Fabbiano et al., 2018).

The effects of protein-restricted diets and caloric restriction on aging were discovered almost simultaneously, and the role of protein restriction in delaying aging was explored. Protein-restricted diets directs the body to reduce the intake of dietary protein or specific amino acids, and dietary protein restriction has beneficial effects on lifespan, in which fibroblast growth factor 21 (FGF21) is an important molecule in its regulation (Le Couteur et al., 2016). A link has been observed between specific amino acid restriction and delayed aging, and methionine has been extensively studied. Methionine is found in animal foods, and methionine restriction (MR) can produce various benefits, such as improved body metabolism and reduced oxidative stress and inflammation (Ables and Johnson, 2017; Wu G. et al., 2020). MR also upregulates H_2_S production, modulates nutrient-sensing pathways, and inhibits cellular senescence, thus affecting aging (Wang S. Y. et al., 2019; Green et al., 2022), so does the impact of MR on the intestinal microbiota have a place in its mechanism of delaying aging? MR intervention in the D-gal-induced mice leads to alterations in the intestinal microbiota compared with the normal methionine-fed group, thus markedly enhancing the relative abundance of *Lachnospiraceae*, *Turicibacter*, *Roseburia*, and *Investinimonas* and increasing the SCFA-producing bacteria. In addition, MR restores gut integrity, reduces LPS leakage, mediated through the intestinal microbiota to prevent age-related degradation of memory functions, alleviates anxiety-like behaviors, and eliminates inflammation and oxidative stress induced by aging in mice (Xu Y. et al., 2022). However, protein restriction leads to inadequate protein intake and skeletal muscle loss problems in the elderly, and appropriate countermeasures need to be developed to avoid malnutrition and reduce the risk of sarcopenia while delaying aging.

Intermittent fasting (IF) refers to a cycle of fasting periods and casual eating periods, including multiple eating regimens, such as time-restricted eating, alternate-day fasting, and every-other-day feeding (EOD) (Anton et al., 2019; Stekovic et al., 2019). Limited pieces of clinical evidence are available for delaying aging in humans, and they mainly focus on weight loss (Anton et al., 2021). Short-term IF in early life causes a remarkable decrease in the age-related pathology of Drosophila, and the mechanism of action does not depend on the TOR pathway, probably by improving intestinal barrier function and maintaining intestinal health to induce long-lasting beneficial effects (Catterson et al., 2018). Fasting-dependent fatty acid oxidation enhances ISC function in young and old mice, enhances intestinal regeneration, and significantly alters their gut microbiota, but these effects vanished after stopping fasting (Mihaylova et al., 2018; Li L. et al., 2020). A comparison was conducted for over 200 phenotypes from different tissues of aged animals fed *ad libitum* or EOD for life to ascertain whether histopathological, physiological, cellular, and molecular aging characteristics developed slower in the EOD group than in controls; large-scale analyses showed that only a minor fraction of characteristics were delayed by EOD independent of the universal slowing of the aging progression (Xie et al., 2017).

In addition, the intake of certain foods may have age-delaying benefits. For example, the functional food Wushen (WS), which is a powdered mixture of 55 foods, interferes with the body’s gut microbiota through a complex composition of compounds in the food to exert anti-aging effects. After feeding WS to SAMP8 mice, the intestinal microbiota of the WS group displayed a marked enhancement in the abundance of *Luminococcus* and *Butyrivella* and a reduction in *Clostridium pullulans*, *Clostridium luminous*, *Bacteroides coelicolor,* and *Investinimonas*, with changes in the metabolic profile, especially for metabolites involved in linoleic acid metabolism and neuroactive ligand–receptor interactions (Zhang et al., 2021). In comparison with the controls, WS effectively alleviated aging-related decreases in food intake, body weight, muscle mass, and adiposity, improved learning and memory abilities, attenuated cognitive decline, and enhanced immune function in mice (Zhang et al., 2021).

### 4.4 Natural compounds

Some of the natural active ingredients present in food and herbal medicine may have an age-delaying effect and have an advantage over drugs in terms of safety, a few drugs target the gut microbiota to delay aging, and the classic anti-aging drug Rapamycin slows aging independent of the gut microbiota (Schinaman et al., 2019). Polyphenols are important natural compounds, which are broadly divided into the four following groups based on their carbon skeleton: phenolic acids, flavonoids, lignans, and stilbenes (Wu et al., 2021). Among the polyphenol monomers related to influencing the aging process, resveratrol has been widely noticed, but its anti-aging mechanism mainly involves the regulation of apoptosis, inhibition of oxidative stress, and enhancement of mitochondrial function, whether it can delay aging by targeting the gut microbiota needs further study (Zhou et al., 2021). Taxifolin (TAX) is a flavonol, and TAX intervention in D-gal-induced mice improves the imbalance of gut microbiota and increases favorable bacterial abundance, including *Enterorhabdus*, *Bifidobacterium clostridium*, and *Parvibacter*, thereby reversing aging-related neuronal damage in brain tissue, reducing spatial learning impairment, improving oxidative stress, and slowing down aging (Liu et al., 2021). Phlorizin (PZ), a flavonoid glycoside found in many botanical fruits and leaves, is of interest for its antioxidant properties (Wang H. et al., 2019). When PZ was administered to D-gal-induced mice, the deleterious effects were reversed, cognitive impairment was delayed, hippocampal neuronal damage was corrected, and the decline in organ coefficients in aging mice was alleviated (Chen et al., 2022). The intestinal microbiota occupies a significant position in its aging-retarding mechanism. PZ modifies the construction and diversity of the intestinal microbiota in mice and corrects aging-related alterations in *Lactobacillus* and *Bacteroides*, and the PZ-induced alterations in bacterial flora metabolites may be engaged in antioxidant and anti-apoptotic effects and thus play a combined anti-aging role (Chen et al., 2022). Lemon polyphenols (LPP), which are mainly found in lemon, interferes with senescence-accelerated mouse prone 1 (SAMP1) to maintain the structural stabilization of its gut microbiota, and LPP increases the GM Firmicutes/Bacteroidetes (F/B) ratio and decreases *Lactobacillus* levels in SAMP1 compared with control SAMP1 mice, thus delaying the increase in senescence-related scores and motoring atrophy through direct or indirect effects of the flora (Shimizu et al., 2019). In addition, various compounds such as Urolithin A (UA) are generated through the metabolism of polyphenols by the intestinal microbiota, and UA levels decrease with age (Cortés-Martín et al., 2018). UA modulates the intestinal microbiota, enhances species richness in HFD-induced obese mice, significantly strengthens the gut barrier, and reduces the inflammatory response in mice (Singh et al., 2019; Ao et al., 2021). UA can promote organismal mitophagy through the PTEN-induced kinase1/Parkin-dependent pathway or directly recruit the microtubule-associated protein LC3 to remove dysfunctional or redundant mitochondria, activate related pathways to protect mitochondrial function, mitigate cellular senescence, and prevent age-related muscle decline (Ryu et al., 2016; Christina et al., 2017; Palikaras et al., 2018; Shi et al., 2021; Liu et al., 2022).

Icariin, the main active ingredient from the Herba Epimedii, has several health benefits. In a previous study, icariin intervention reduced the alpha diversity and intragroup variability of the intestinal microbiota in aged mice, causing a younger microbiota, lower abundance of *Alistipes*, *Akkermansia,* and *Butyrivibrio*, and higher *Mucispirillum* in older mice, and it also maintained gut epithelial integrity (Li et al., 2021). In comparison with aged controls, icariin intervention enhanced mobile learning and coordination in aged mice, inhibited aging-induced oxygen radical damage, and upregulated mRNAs of aging-related genes to levels similar to those of young mice, including Sirt 1, 3, and 6 and the telomere binding protein (Li et al., 2021). In the FMT experiment, feces from icariin-intervened aged mice were transplanted to normal aged mice, and aging-related characteristics were improved in the recipient mice, although the changes were not as significant as direct icariin intervention (Li et al., 2021)*. Rhododendron nivale Hook.f* (*R.n*) in Tibetan medicine has various pharmacological effects, and its ethanolic extract residues (RNEA) mainly includes hyperin and diplomorphanin B, etc. RNEA treatment significantly improved age-related memory loss in mice by remodeling gut flora and mediating enhanced antioxidant capacity, and glutathione peroxidase activity was similarly enhanced by transferring the fecal microbiota from RNEA-treated mice to GF mice (Guo et al., 2022).

## 5 Discussion

Both the intestinal microbiota and aging are sophisticated subjects. The human intestinal microbiota undergoes significant changes during aging, and it is closely related to aging. However, the causal debate between intestinal microbiota and aging continues, and the analysis results indicate that they co-evolve and are mutually causal. The study of aging through the gut microbiota is a promising direction, whether it is to target the intestinal microbiota for intervention or to explore the underlying mechanisms of aging. Interventions to delay aging primarily aim at aging drivers. Several animal studies have confirmed that aging can be delayed by FMT, probiotics, diet, and other regulation of the gut microbiota. However, the specific microbial characteristics related to delayed aging and maintenance of youth still need to be combined with several related experimental results for professional summary analysis. One researcher found a higher abundance of favorable bacteria in all of the experimental mice coexisting in the young trait-associated gut microbiota by Euler–Venn analysis (Li et al., 2021). Human-delayed aging trials should be considered in many aspects, focusing on overall variations in the intestinal microbiota. Lifelong monitoring of the microbiota can be considered. A relevant platform needs to be developed to achieve big data integration and efficient use of experimental data, reliable age biomarkers should also be optimized, a perfect system for the assessment of biological aging should be established, personalized interventions should be emphasized, and precise interventions should be advocated. The intrinsic links between the various aging drivers need to be focused on, multiple pathways need be combined to achieve anti-aging goals, and the right time to intervene needs to be determined immediately before the emergence of age-related diseases. The follow-up on delayed aging also needs attention. Will it lead to a shorter or longer life span of the organism? Will it remain young until natural death? Moreover, considering the high complexity of the intestinal microbiota, the relevant research techniques still need to be upgraded and optimized at this stage. Only by fully understanding the gut microbiota can we uncover deeper mysteries, with the aim of early understanding of its complex relationship with host aging.

## Author contributions

YZ: Writing – original draft. XW: Writing – original draft. WL: Writing – original draft. YY: Writing – original draft. ZW: Writing – original draft. YL: Writing – review & editing. CY: Writing – review & editing.
